# A Bioengineered Neuregulin-Hydrogel Therapy Reduces Scar Size and Enhances Post-Infarct Ventricular Contractility in an Ovine Large Animal Model

**DOI:** 10.3390/jcdd7040053

**Published:** 2020-11-17

**Authors:** Jeffrey E. Cohen, Andrew B. Goldstone, Hanjay Wang, Brendan P. Purcell, Yasuhiro Shudo, John W. MacArthur, Amanda N. Steele, Michael J. Paulsen, Bryan B. Edwards, Chiaka N. Aribeana, Nicholas C. Cheung, Jason A. Burdick, Y. Joseph Woo

**Affiliations:** 1 Department of Cardiothoracic Surgery, Stanford University, Stanford, CA 94305, USA; jeffrey.e.cohen@medstar.net (J.E.C.); goldstonea@email.chop.edu (A.B.G.); hanjay@stanford.edu (H.W.); yshudo@stanford.edu (Y.S.); jwm2108@stanford.edu (J.W.M.); ansteele@alumni.stanford.edu (A.N.S.); mpaulsen@stanford.edu (M.J.P.); bryan.b.edwards@gmail.com (B.B.E.); chiaka.aribeana@gmail.com (C.N.A.); ncheung14@gmail.com (N.C.C.); 2 Department of Bioengineering, University of Pennsylvania, Philadelphia, PA 19104, USA; brendan.p.purcell@gmail.com (B.P.P.); burdick2@seas.upenn.edu (J.A.B.); 3 Department of Bioengineering, Stanford University, Stanford, CA 94305, USA

**Keywords:** neuregulin, hydrogel, myocardial infarction, ischemic heart failure

## Abstract

The clinical efficacy of neuregulin (NRG) in the treatment of heart failure is hindered by off-target exposure due to systemic delivery. We previously encapsulated neuregulin in a hydrogel (HG) for targeted and sustained myocardial delivery, demonstrating significant induction of cardiomyocyte proliferation and preservation of post-infarct cardiac function in a murine myocardial infarction (MI) model. Here, we performed a focused evaluation of our hydrogel-encapsulated neuregulin (NRG-HG) therapy’s potential to enhance cardiac function in an ovine large animal MI model. Adult male Dorset sheep (*n* = 21) underwent surgical induction of MI by coronary artery ligation. The sheep were randomized to receive an intramyocardial injection of saline, HG only, NRG only, or NRG-HG circumferentially around the infarct borderzone. Eight weeks after MI, closed-chest intracardiac pressure–volume hemodynamics were assessed, followed by heart explant for infarct size analysis. Compared to each of the control groups, NRG-HG significantly augmented left ventricular ejection fraction (*p* = 0.006) and contractility based on the slope of the end-systolic pressure–volume relationship (*p* = 0.006). NRG-HG also significantly reduced infarct scar size (*p* = 0.002). Overall, using a bioengineered hydrogel delivery system, a one-time dose of NRG delivered intramyocardially to the infarct borderzone at the time of MI in adult sheep significantly reduces scar size and enhances ventricular contractility at 8 weeks after MI.

## 1. Introduction

Coronary disease represents an immense global health challenge, accounting for nearly 9 million deaths worldwide each year [1], and is projected to increase in treatment cost by nearly 100% by the year 2030 [2]. While improvements in medical and procedural therapies have undoubtedly decreased the morbidity and mortality associated with myocardial infarction (MI), there remains a dire need for advanced therapeutics in mitigating the progression to heart failure following acute ischemic events. Considerable progress has been achieved through a range of strategies including cell therapy [3,4], immunomodulation [5], tissue engineering [6,7], angiogenic cytokines [8,9], and direct oxygen synthesis [10,11]. Other recent studies have highlighted the intrinsic neovasculogenic and regenerative capacity of neonatal mammalian hearts [12,13,14], and have also illustrated that human adult myocardium retains some proliferative potential [15]. Our group and others have built upon the strategy of reactivating regenerative mechanisms after MI and previously established that exogenous administration of neuregulin-1β (NRG) induces cardiomyocyte proliferation and improves cardiac function in small and large animal models of MI and ischemic cardiomyopathy [16,17,18].

NRG is a member of the epidermal growth factor family and is an important director of embryonic cardiac development [19]. Specifically, NRG is a ligand to the ErbB4 receptor expressed by cardiomyocytes [20]. Binding of NRG to ErbB4 results in heterodimerization of ErbB4 with ErbB2, which then activates tyrosine kinase-mediated signaling pathways, including extracellular signal-regulated kinase (ERK) [21,22]. An initial clinical finding that suggested the potential benefits of NRG administration was the depressive cardiac effects of the breast cancer drug trastuzumab, in which the inhibition of the ErbB4 tyrosine kinase pathway led to heart failure in some patients [23]. This observation suggested that NRG’s activation of this pathway could yield cardioprotective effects [24].

The strong mechanistic understanding of NRG’s cardioprotective effects has led to clinical trials using daily systemic NRG infusions for patients with chronic heart failure. The results of these trials, however, have demonstrated only modest benefits [25,26], although a phase III human clinical trial using a recombinant NRG fragment is currently recruiting patients and remains in progress (NCT03388593). A major drawback of this parenteral therapy is the requirement of daily repetitive dosing. More recently, a phase I human clinical trial using a glycosylated isotype of recombinant NRG in a single intravenous dose in 40 patients with left ventricular systolic dysfunction was completed, showing a sustained improvement in cardiac function for up to 3 months [27]. Another strategy that has recently been investigated utilized subcutaneous NRG injections (NCT01214096). 

All of these methods, however, employ systemic or otherwise untargeted delivery, leading to a potentially suboptimal therapeutic concentration of NRG in the heart. Our team has endeavored to circumvent these obstacles by utilizing engineered hydrogels (HG) to permit targeted and sustained one-time delivery of therapeutic agents directly to the myocardium [28,29,30]. Within the hydrogel, a therapeutic agent may be protected from degradation to increase its half-life, kept from diffusing away from the target site to maintain a greater effective concentration, and gradually released to prolong the duration of exposure beyond a bolus dose. Indeed, we previously demonstrated that NRG encapsulated within a HG could be released gradually over a course of 14 days, and that intramyocardial administration of this hydrogel-encapsulated neuregulin (NRG-HG) therapy at the time of MI in adult mice significantly increased cardiomyocyte survival and proliferation, and enhanced post-MI ventricular function [18].

Based on our previous success in implementing HG-encapsulated NRG in a small animal MI model, the current study serves as a preliminary, focused assessment of cardiac function associated with NRG-HG therapy in a large animal MI model. We hypothesized that NRG-HG reduces infarct scar size and enhances ventricular contractility after MI in an ovine model. 

## 2. Materials and Methods 

### 2.1. Study Design

This four-arm study was conducted specifically to evaluate the potential of HG-encapsulated NRG to enhance cardiac function in a large animal MI model, compared to controls receiving sterile saline, HG alone, or NRG alone. Ejection fraction and the slope of the end-systolic pressure–volume relationship (ESPVR) were the primary outcomes of interest. All experiments pertaining to this investigation were fully blinded and conformed to the “Guide for the Care and Use of Laboratory Animals,” published by the United States National Institutes of Health [31]. The protocol was approved by the Administrative Panel on Laboratory Animal Care of Stanford University (#28943, 2014–2020). 

### 2.2. Macromer Synthesis, Hydrogel Formation, and Neuregulin Encapsulation

The techniques used in this study for macromer synthesis, hydrogel formation, and NRG encapsulation have been previously described [18]. Briefly, sodium hyaluronate (74 kDa, Lifecore Biomedical Inc., Chaska, MN, USA) was chemically modified with hydroxyethyl methacrylate (HEMA) to introduce a terminal methacrylate group for free radical-initiated crosslinking, and with ester bonds to permit hydrolytic degradation [32]. This process was achieved by reacting HEMA with succinic anhydride via a ring-opening polymerization in the presence of N-methylimidazole to yield HEMA-COOH, which was then coupled to a tetrabutylammonium salt of hyaluronic acid (HA) in the presence of 4-dimethylaminopyridine to produce HA macromers with the HEMA group modification (HEMA-HA). After purification via dialysis and lyophilization, approximately 15% of HA disaccharides were modified with a HEMA group, based on ^1^H nuclear magnetic resonance.

HG formation was achieved using a two-component redox initiator system, in which ammonium persulfate and *N*,*N*,*N*′,*N*′-tetramethylethylenediamine were added to a final concentration of 10 mM in a 4% (*w*/*v*) HEMA-HA solution on ice. The HG gelation kinetics were characterized via rheometry (Texas Instruments AR 2000ex, Dallas, TX, USA) at 37 °C by assessing the storage (G’) and loss (G”) modulus over time, while applying oscillatory strain (20 mm 1° cone geometry, 1% strain, 1 Hz). 

Recombinant human NRG-1β EGF domain (Thr176-Lys246, R&D Systems, Minneapolis, MN, USA) was used in this study. HG encapsulation of NRG was achieved by adding 100 µg of NRG to 1 mL of the HG precursor solution described above. 

### 2.3. Ovine Myocardial Infarction Model

Healthy adult (6–7 month old) male Dorset sheep weighing approximately 35–40 kg (*n* = 21, Pozzi Ranch, Sonoma, CA, USA) underwent surgical induction of acute MI using well-established and reproducible techniques of ovine anesthesia and surgery [8,30,33,34]. Each sheep received 600 mg of oral amiodarone daily for 72 h prior to surgery. After fasting the night before surgery, the sheep were sedated with intramuscular ketamine (4–10 mg/kg) and diazepam (0.05–0.2 mg/kg), followed by intravenous propofol (5–12 mg/kg). Next, the sheep were intubated, and general anesthesia was maintained using 2% isoflurane for the duration of the procedure. Intravenous hydromorphone (0.01–0.025 mg/kg) was administered for perioperative analgesia, and cefazolin (20 mg/kg) was given for perioperative antibiotic prophylaxis. After undergoing sterile percutaneous placement of an arterial line via the right femoral artery and a central line via the right internal jugular vein, the animals were then placed in right lateral decubitus position and sterilely prepped and draped. Intravenous infusions of lidocaine (10–50 µg/kg/min) and amiodarone (0.08–0.12 mg/kg/min) were initiated. 

**Figure 1:**
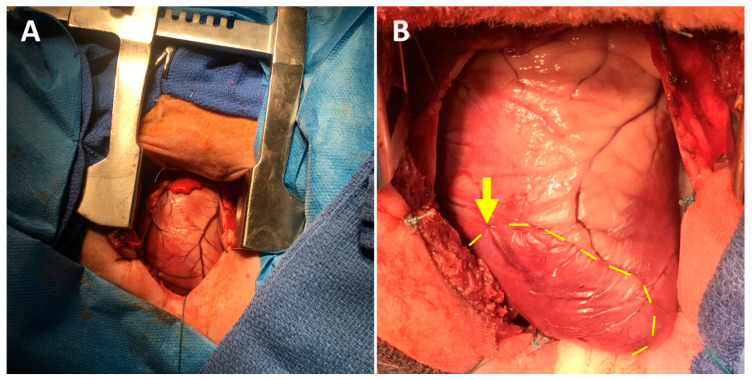
Ovine myocardial infarction model using a mini left thoracotomy approach. (**A**) The left ventricle is exposed through a fifth interspace mini left thoracotomy incision. (**B**) Diagonal branches of the left anterior descending coronary artery are ligated using 5-0 or 6-0 polypropylene sutures (arrow), producing a consistently-sized pale and hypokinetic infarct zone (bounded by dotted line). The infarct borderzone lies immediately beyond the dotted yellow line.

A 5 cm minimally invasive left thoracotomy incision was made (Figure 1A). The chest was entered through the fifth intercostal space, and the pericardium was opened to expose the left ventricle (LV). Next, the second and third diagonal branches of the left anterior descending coronary artery were ligated using 5-0 or 6-0 polypropylene sutures (Figure 1B). Prior to permanently tying the ligation stitches, the sutures were snared and the area of resulting pallor and hypokinesis was assessed to ensure a consistent and uniform 30% LV infarct. Electrocardiographic ST-segment elevation changes were observed in all cases. Ten minutes after permanently inducing the MI, the animals were randomized to receive intramyocardial injections of either sterile saline, HG alone, NRG alone, or HG-encapsulated NRG. In all cases, the treatments consisted of a total volume of 1 mL divided into ten 100 µL aliquots, administered to the LV myocardium using 28 G insulin syringe needles. The aliquots were injected circumferentially around the infarct borderzone, which was defined as the perfused myocardium immediately adjacent to the pale and hypokinetic ischemic territory. Importantly, the surgeon was blinded at all times to the experimental groups. Following treatment, the chest was closed in layers, the pneumothorax was evacuated with a temporary chest tube, and the animal was finally extubated and recovered. Intramuscular buprenorphine (0.006 mg/kg) was administered for postoperative analgesia. 

The sheep were housed singly for one week after surgery to monitor for adequate pain control, wound healing, activity, food intake, weight gain, urination, and defecation. Afterwards, all sheep were housed in pairs. Food and water were provided ad libitum after surgery.

### 2.4. Intracardiac Hemodynamics Testing and Heart Explant

After 8 weeks, all sheep underwent terminal surgery using the same protocol for anesthesia and analgesia as described above. After intubation, the sheep were positioned in right lateral decubitus position. A 3 cm incision was made in the left neck to isolate the left carotid artery and the left internal jugular vein. A 7 Fr sheath was inserted into the carotid artery and a 9 Fr sheath was inserted into the jugular vein. For closed-chest hemodynamics assessment, a 5 Fr pressure–volume loop catheter (Ventri-Cath 510, Millar, Houston, TX, USA) was inserted via the carotid artery sheath and positioned in the LV using fluoroscopic guidance. Preload-independent measurements were acquired during occlusion of the inferior vena cava using an 8–14 Fr Fogarty balloon inserted via the internal jugular vein sheath and positioned in the supra-hepatic inferior vena cava using fluoroscopic guidance. Subsequently, a median sternotomy was performed and intravenous potassium chloride (1 mEq/kg) was administered to induce cardiac arrest. Finally, the heart was explanted. 

### 2.5. Infarct Size Analysis

Following cardiac explant, the atria were removed, and the LV was opened along the posterior septum. Standardized orthogonal digital photographs were taken of the LV endocardium (Nikon D5100 SLR camera, Tokyo, Japan). Photographs were uploaded to ImageJ (version 1.52a, National Institutes of Health, Bethesda, MD, USA) and the size of the infarct was assessed using digital planimetry in a blinded fashion. 

### 2.6. Statistical Analyses

Statistical analyses were performed using one-way analysis of variance with Tukey’s test for post-hoc comparisons (Stata version 14.2, StataCorp LLC., College Station, TX, USA). Data are reported as mean with standard error. A p-value less than 0.05 was considered statistically significant. 

## 3. Results

### 3.1. Hydrogel-Encapsulated Neuregulin Enhances Post-Infarct Cardiac Function and Contractility

**Figure 2:**
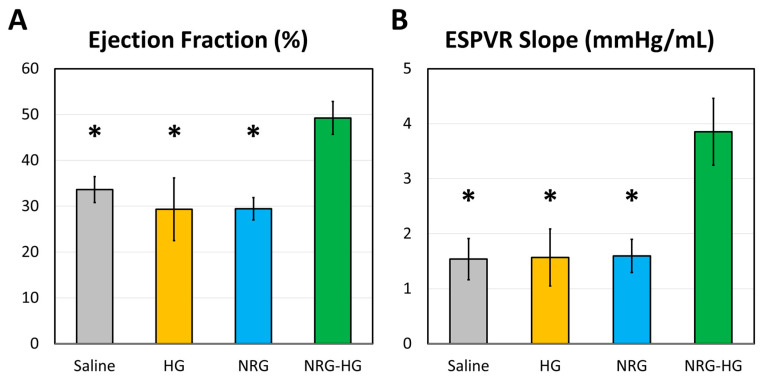
Cardiac function at 8 weeks after myocardial infarction, measured by analyzing closed-chest left ventricular pressure-volume hemodynamics data. Data regarding (**A**) ejection fraction (%) and (**B**) the slope of the end-systolic pressure–volume relationship (ESPVR, mmHg/mL) are compared among sheep receiving saline (*n* = 6), hydrogel only (HG, *n* = 4), neuregulin only (NRG, *n* = 4), and hydrogel-encapsulated neuregulin (NRG-HG, *n* = 6). Data are presented as mean ± standard error. * indicates statistically significant (*p* < 0.05) compared to the NRG-HG group.

**Figure 3:**
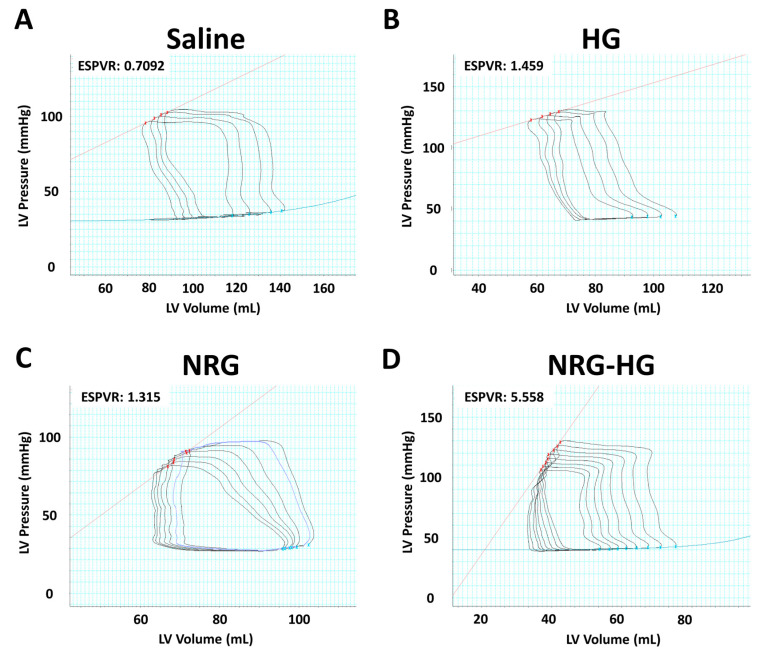
Representative pressure–volume loops for the (**A**) saline, (**B**) hydrogel only (HG), (**C**) neuregulin only (NRG), and (**D**) hydrogel-encapsulated neuregulin (NRG-HG) groups are shown. The slope of the end-systolic pressure-volume relationship (ESPVR, red line) is quantified.

At 8 weeks after MI, the sheep in the saline (*n* = 6), HG (*n* = 4), NRG (*n* = 4), and NRG-HG groups (*n* = 7) all exhibited similar heart rate (*p* = 0.290) and blood pressure (*p* = 0.835). Closed-chest pressure–volume hemodynamics data were successfully obtained for all except one NRG-HG sheep (Table 1). In terms of cardiac geometry, a trend was observed for sheep in the NRG-HG group to have smaller LV volumes, both during diastole (saline 108.9 vs. HG 102.8 vs. NRG 116.3 vs. NRG-HG 79.1 mL, *p* = 0.182) and systole (saline 73.9 vs. HG 77.3 vs. NRG 83.1 vs. NRG-HG 43.1 mL, *p* = 0.077), although these differences did not reach statistical significance. After normalizing for body size [35], the sheep in the NRG-HG group had a significantly enhanced stroke work index (saline 1.72 vs. HG 1.22 vs. NRG 1.24 vs. NRG-HG 2.67 mmHg·L/m^2^, *p* = 0.003) and cardiac power index (saline 0.35 vs. HG 0.27 vs. NRG 0.22 vs. NRG-HG 0.55 W/m^2^, *p* = 0.014). Finally, the sheep in the NRG-HG group also exhibited significantly augmented ejection fraction (saline 33.6% vs. HG 29.4% vs. NRG 29.4% vs. NRG-HG 49.3%, *p* = 0.006, Figure 2A) and LV contractility based on the slope of the ESPVR (saline 1.54 vs. HG 1.57 vs. NRG 1.60 vs. NRG-HG 3.85 mmHg/mL, *p* = 0.006, Figure 2B). Representative pressure–volume loops with quantified ESPVR slope are presented in Figure 3. 

### 3.2. Hydrogel-Encapsulated Neuregulin Reduces Infarct Size

**Figure 4:**
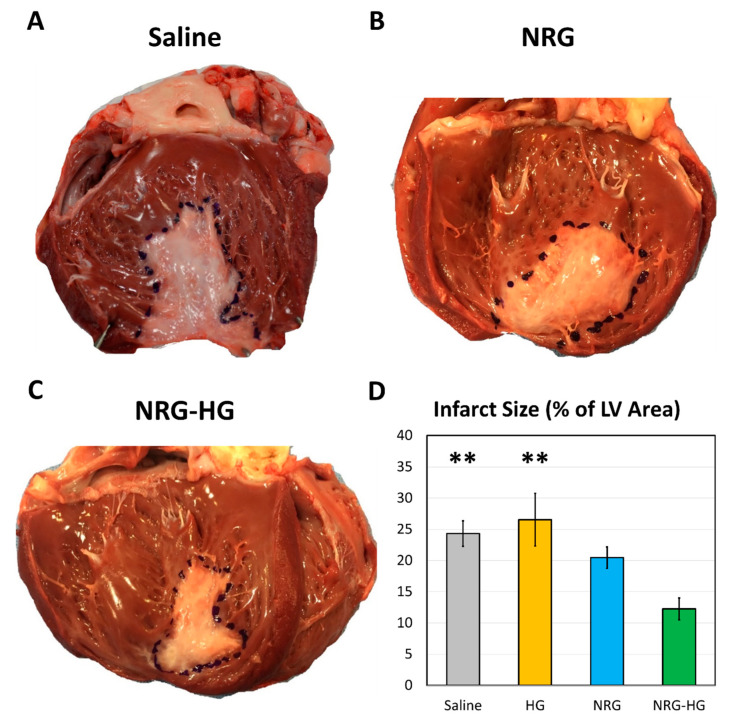
Left ventricular endocardial scar size at 8 weeks after myocardial infarction. Representative photographs of sheep hearts from the (**A**) saline, (**B**) neuregulin only (NRG), and (**C**) hydrogel-encapsulated neuregulin (NRG-HG) groups are shown. (**D**) Quantified scar size for the saline (*n* = 6), hydrogel only (HG, *n* = 4), NRG only (*n* = 4), and NRG-HG groups (*n* = 7). Data are presented as mean ± standard error. ** indicates statistically significant (*p* < 0.01) compared to the NRG-HG group.

All hearts were explanted at 8 weeks after MI, following completion of hemodynamics and cardiac function testing. A clearly delineated region of fibrotic scar formation with associated thinning of the infarcted LV was identified in all sheep. Relative to the endocardial surface area of the LV, the size of the infarct scar was smallest among the sheep in the NRG-HG group (saline 24.3% vs. HG 26.5% vs. NRG 20.5% vs. NRG-HG 12.3%, *p* = 0.002, Figure 4). 

## 4. Discussion

In this fully-blinded, four-arm ovine large animal study, we focused on investigating the potential of HG-encapsulated NRG to preserve cardiac function after MI, as this data represents an essential preliminary step in determining the suitability of advancing HG-based NRG cardiac delivery to the clinical arena. We demonstrated that intramyocardial borderzone injection of NRG-HG therapy at the time of MI in adult sheep significantly reduces infarct scar size and enhances post-infarct LV contractility, thus mitigating the progression to ischemic heart failure after MI. 

Cardiac function was measured using closed-chest pressure–volume hemodynamics analyses. Specifically, ejection fraction was selected as a primary outcome of interest because of its strong association with mortality in patients with heart failure [36]. Indeed, in humans, below a threshold ejection fraction of 45%, the hazard ratio of all-cause mortality dramatically increases by 39% for every 10% decrease in ejection fraction. At an ejection fraction above 45%, however, the risk of mortality remains relatively stable [36]. Although no animal model perfectly replicates human cardiac physiology, ovine hearts exhibit remarkable similarities in terms of molecular and cellular organization, cardiomyocyte functional properties, and overall hemodynamics [37]. In our study, the hearts of sheep in the NRG-HG group demonstrated a median ejection fraction of 49.3%, whereas the median ejection fraction of sheep in the various control groups ranged from 29.4% to 33.6%. This finding that our NRG-HG therapy significantly augments ejection fraction in an ovine model aligns with the observed improvement in ejection fraction among patients with heart failure who received NRG in clinical trials [25,26].

In addition, we also used the slope of the ESPVR as a primary outcome of interest in this study. Unlike ejection fraction, which is dependent on both preload and afterload [38], the ESPVR slope reflects LV contractility independent of preload and afterload, and is also resistant to changes in heart rate as well [39,40,41]. In agreement with our data for ejection fraction, the sheep in the NRG-HG group exhibited an ESPVR slope over two times that of the various control groups. This result confirms the significant therapeutic potential of our NRG-HG therapy to preserve cardiac function after MI. 

Along with improvements in functional performance, the NRG-HG therapy was also found to significantly reduce infarct size. The large infarct scar observed in the saline control group (24.3% of the LV endocardial surface area) demonstrates the successful induction of MI in this ovine model. Furthermore, the similarity in scar size between the saline group and non-therapeutic HG-only group (26.5% of LV endocardial area) attests to the reproducibility of our technique. As expected, the sheep in the NRG-only group exhibited a slightly reduced scar size (20.5% of LV endocardial area), but this small degree of recovery was inadequate for the preservation of cardiac function. Perhaps as a result of targeted and sustained delivery of NRG via our HG-mediated delivery system, the sheep in the NRG-HG group had remarkably small infarct scars (12.3% of LV endocardial area). In the current clinical arena, all-cause mortality and hospital admissions for patients undergoing percutaneous coronary intervention after MI are strongly associated with infarct size [42], thus further suggesting that the benefits of our NRG-HG therapy may potentially be translatable to clinical practice. 

There are a number of limitations to this study. First, given that NRG’s ability to regenerate the myocardium and the mechanism of action are both well-described by a large body of scientific literature [16,22], and because this is a large animal study requiring four experimental arms, we determined that it was not feasible from an ethical standpoint to nearly double the number of animals sacrificed in this study to re-validate the molecular mechanism of this NRG therapy at another post-MI timepoint. Thus, this work represents a purely functional study, and is not designed to acquire mechanistic data either through molecular assays or histological analyses. Related to this limitation, it is likely that additional statistically significant results would be realized with larger sample sizes, especially with regards to post-MI LV remodeling. The addition of imaging studies (e.g., using echocardiography) may also be useful in this regard. However, because the current study, in a completely blinded fashion, was able to demonstrate statistically significant differences among our primary functional outcomes of interest, as well as in infarct size, we elected not to subject more large animals to experimentation. Furthermore, although we did not measure the ischemic area immediately after coronary ligation for each sheep, the sheep were randomized to each treatment group and the surgeons remained blinded to the treatment identity at all times, precluding the introduction of selection bias. A final limitation is that the injections were performed immediately after acute infarction and using a thoracotomy, which does not accurately represent the clinical scenario, in which intervention, often percutaneous catheter-based revascularization, is delayed by the time needed for presentation to medical attention. As hydrogels and other biomaterial technologies continue to rapidly evolve, such as with novel shear-thinning hydrogels [29,30,43], it may be possible to use percutaneous catheter-based approaches or alternate materials to address this concern. The delay in intervention in real world practice may also result in differences in myocardial survival compared to our laboratory data. Despite these limitations, our current study serves as a preliminary demonstration of the ability of HG-encapsulated NRG to significantly enhance cardiac function and reduce scar size in a large animal MI model, thus establishing an essential foundation for future preclinical studies. 

## 5. Conclusions

Using a bioengineered hydrogel delivery system, a one-time dose of NRG delivered intramyocardially to the infarct borderzone at the time of MI in adult sheep significantly reduces scar size and enhances cardiac function at 8 weeks after MI. 

## Figures and Tables

**Table 1:** Hemodynamics and cardiac function at 8 weeks after myocardial infarction

	Saline (*n* = 6)	HG (*n* = 4)	NRG (*n* = 4)	NRG-HG (*n* = 7)	ANOVA *p*-Value
Heart Rate (bpm)	91.2 ± 2.6 *p* = 1.000	92.4 ± 10.3 *p* = 0.998	79.0 ± 2.9 *p* = 0.340	91.1 ± 3.6	0.290
Mean Arterial Pressure (mmHg)	71.5 ± 4.1 *p* = 0.898	68.8 ± 13.6 *p* = 0.838	72.8 ± 15.8 *p* = 0.954	79.3 ± 5.4	0.835
End-Diastolic Volume (mL)	108.9 ± 11.0 *p* = 0.279	102.8 ± 20.8 *p* = 0.559	116.3 ± 6.3 *p* = 0.200	79.1 ± 9.8 ^a^	0.182
End-Systolic Volume (mL)	73.9 ± 8.3 *p* = 0.186	77.3 ± 21.9 *p* = 0.191	83.1 ± 4.5 *p* = 0.103	43.1 ± 8.1 ^a^	0.077
Stroke Work Index (mmHg·L/m^2^)	1.72 ± 0.14 *p* = 0.053	1.22 ± 0.36 *p* = 0.007 *	1.24 ± 0.21 *p* = 0.008 *	2.67 ± 0.31 ^a^	0.003 *
Cardiac Power Index (W/m^2^)	0.35 ± 0.02 *p* = 0.134	0.27 ± 0.11 *p* = 0.047 *	0.22 ± 0.04 *p* = 0.018 *	0.55 ± 0.09 ^a^	0.014 *
Ejection Fraction (%)	33.6 ± 2.8 *p* = 0.034 *	29.4 ± 6.8 *p* = 0.015 *	29.4 ± 2.4 *p* = 0.015 *	49.3 ± 3.6 ^a^	0.006 *
Slope of ESPVR (mmHg/mL)	1.54 ± 0.38 *p* = 0.011 *	1.57 ± 0.52 *p* = 0.026 *	1.60 ± 0.30 *p* = 0.028 *	3.85 ± 0.61 ^a^	0.006 *

Hemodynamics and cardiac function are compared among sheep receiving saline, hydrogel only (HG), neuregulin only (NRG), and hydrogel-encapsulated neuregulin (NRG-HG). Data are presented as mean ± standard error. ^a^ Data available for *n* = 6. * indicates statistically significant (*p* < 0.05). The groups were compared by one-way analysis of variance (ANOVA). *p*-values of Tukey’s post-hoc test are presented relative to the NRG-HG group. ESPVR, end-systolic pressure–volume relationship.

## References

[1] Virani S.S., Alonso A., Benjamin E.J., Bittencourt M.S., Callaway C.W., Carson A.P., Chamberlain A.M., Chang A.R., Cheng S., Delling F.N. (2020). American Heart Association Council on Epidemiology and Prevention Statistics Committee and Stroke Statistics Subcommittee Heart Disease and Stroke Statistics-2020 Update: A Report From the American Heart Association. Circulation.

[2] Heidenreich P.A., Trogdon J.G., Khavjou O.A., Butler J., Dracup K., Ezekowitz M.D., Finkelstein E.A., Hong Y., Johnston S.C., Khera A. (2011). Forecasting the future of cardiovascular disease in the United States: A policy statement from the American Heart Association. Circulation.

[3] Ascheim D.D., Gelijns A.C., Goldstein D., Moye L.A., Smedira N., Lee S., Klodell C.T., Szady A., Parides M.K., Jeffries N.O. (2014). Mesenchymal precursor cells as adjunctive therapy in recipients of contemporary left ventricular assist devices. Circulation.

[4] Menasché P. (2018). Cell therapy trials for heart regeneration—Lessons learned and future directions. Nat. Rev. Cardiol..

[5] Luger D., Lipinski M.J., Westman P.C., Glover D.K., Dimastromatteo J., Frias J.C., Albelda M.T., Sikora S., Kharazi A., Vertelov G. (2017). Intravenously Delivered Mesenchymal Stem Cells: Systemic Anti-Inflammatory Effects Improve Left Ventricular Dysfunction in Acute Myocardial Infarction and Ischemic Cardiomyopathy. Circ. Res..

[6] Wanjare M., Kawamura M., Hu C., Alcazar C., Wang H., Woo Y.J., Huang N.F. (2019). Vascularization of Engineered Spatially Patterned Myocardial Tissue Derived From Human Pluripotent Stem Cells in vivo. Front. Bioeng. Biotechnol..

[7] Von Bornstädt D., Wang H., Paulsen M.J., Goldstone A.B., Eskandari A., Thakore A., Stapleton L., Steele A.N., Truong V.N., Jaatinen K. (2018). Rapid Self-Assembly of Bioengineered Cardiovascular Bypass Grafts From Scaffold-Stabilized, Tubular Bilevel Cell Sheets. Circulation.

[8] Macarthur J.W., Cohen J.E., McGarvey J.R., Shudo Y., Patel J.B., Trubelja A., Fairman A.S., Edwards B.B., Hung G., Hiesinger W. (2014). Preclinical evaluation of the engineered stem cell chemokine stromal cell-derived factor 1α analog in a translational ovine myocardial infarction model. Circ. Res..

[9] Wang H., Wisneski A., Paulsen M.J., Imbrie-Moore A., Wang Z., Xuan Y., Hernandez H.L., Lucian H.J., Eskandari A., Thakore A.D. (2019). Bioengineered analog of stromal cell-derived factor 1α preserves the biaxial mechanical properties of native myocardium after infarction. J. Mech. Behav. Biomed. Mater..

[10] Cohen J.E., Goldstone A.B., Paulsen M.J., Shudo Y., Steele A.N., Edwards B.B., Patel J.B., MacArthur J.W., Hopkins M.S., Burnett C.E. (2017). An innovative biologic system for photon-powered myocardium in the ischemic heart. Sci. Adv..

[11] Wang H., Wu M.A., Woo Y.J. (2019). Photosynthetic symbiotic therapy. Aging (Albany N. Y.).

[12] Das S., Goldstone A.B., Wang H., Farry J., D’Amato G., Paulsen M.J., Eskandari A., Hironaka C.E., Phansalkar R., Sharma B. (2019). A unique collateral artery development program promotes neonatal heart regeneration. Cell.

[13] Wang H., Paulsen M.J., Hironaka C.E., Shin H.S., Farry J.M., Thakore A.D., Jung J., Lucian H.J., Eskandari A., Anilkumar S. (2020). Natural heart regeneration in a neonatal rat myocardial infarction model. Cells.

[14] Wang H., Bennett-Kennett R., Paulsen M.J., Hironaka C.E., Thakore A.D., Farry J.M., Eskandari A., Lucian H.J., Shin H.S., Wu M.A. (2020). Multiaxial Lenticular Stress-Strain Relationship of Native Myocardium is Preserved by Infarct-Induced Natural Heart Regeneration in Neonatal Mice. Sci. Rep..

[15] Bergmann O., Bhardwaj R.D., Bernard S., Zdunek S., Barnabé-Heider F., Walsh S., Zupicich J., Alkass K., Buchholz B.A., Druid H. (2009). Evidence for cardiomyocyte renewal in humans. Science.

[16] Bersell K., Arab S., Haring B., Kühn B. (2009). Neuregulin1/ErbB4 signaling induces cardiomyocyte proliferation and repair of heart injury. Cell.

[17] Liu X., Gu X., Li Z., Li X., Li H., Chang J., Chen P., Jin J., Xi B., Chen D. (2006). Neuregulin-1/erbB-activation improves cardiac function and survival in models of ischemic, dilated, and viral cardiomyopathy. J. Am. Coll. Cardiol..

[18] Cohen J.E., Purcell B.P., MacArthur J.W., Mu A., Shudo Y., Patel J.B., Brusalis C.M., Trubelja A., Fairman A.S., Edwards B.B. (2014). A bioengineered hydrogel system enables targeted and sustained intramyocardial delivery of neuregulin, activating the cardiomyocyte cell cycle and enhancing ventricular function in a murine model of ischemic cardiomyopathy. Circ. Heart Fail.

[19] Wadugu B., Kühn B. (2012). The role of neuregulin/ErbB2/ErbB4 signaling in the heart with special focus on effects on cardiomyocyte proliferation. Am. J. Physiol. Heart Circ. Physiol..

[20] Zhao Y., Sawyer D.R., Baliga R.R., Opel D.J., Han X., Marchionni M.A., Kelly R.A. (1998). Neuregulins promote survival and growth of cardiac myocytes. J. Bio. Chem..

[21] Citri A., Yarden Y. (2006). EGF-ERBB signalling: Towards the systems level. Nat. Rev. Mol. Cell Biol..

[22] Odiete O., Hill M.F., Sawyer D.B. (2012). Neuregulin in cardiovascular development and disease. Circ. Res..

[23] Jerian S., Keegan P. (1999). Cardiotoxicity associated with paclitaxel/trastuzumab combination therapy. J. Clin. Oncol..

[24] Lemmens K., Doggen K., De Keulenaer G.W. (2007). Role of neuregulin-1/ErbB signaling in cardiovascular physiology and disease: Implications for therapy of heart failure. Circulation.

[25] Gao R., Zhang J., Cheng L., Wu X., Dong W., Yang X., Li T., Liu X., Xu Y., Li X. (2010). A Phase II, randomized, double-blind, multicenter, based on standard therapy, placebo-controlled study of the efficacy and safety of recombinant human neuregulin-1 in patients with chronic heart failure. J. Am. Coll. Cardiol..

[26] Jabbour A., Hayward C.S., Keogh A.M., Kotlyar E., McCrohon J.A., England J.F., Amor R., Liu X., Li X.Y., Zhou M.D. (2011). Parenteral administration of recombinant human neuregulin-1 to patients with stable chronic heart failure produces favourable acute and chronic haemodynamic responses. Eur. J. Heart Fail..

[27] Lenihan D.J., Anderson S.A., Lenneman C.G., Brittain E., Muldowney J.A.S., Mendes L., Zhao P.Z., Iaci J., Frohwein S., Zolty R. (2016). A Phase I, Single Ascending Dose Study of Cimaglermin Alfa (Neuregulin 1β3) in Patients With Systolic Dysfunction and Heart Failure. JACC Basic Transl. Sci..

[28] MacArthur J.W., Purcell B.P., Shudo Y., Cohen J.E., Fairman A., Trubelja A., Patel J., Hsiao P., Yang E., Lloyd K. (2013). Sustained release of engineered stromal cell-derived factor 1-α from injectable hydrogels effectively recruits endothelial progenitor cells and preserves ventricular function after myocardial infarction. Circulation.

[29] Steele A.N., Stapleton L.M., Farry J.M., Lucian H.J., Paulsen M.J., Eskandari A., Hironaka C.E., Thakore A.D., Wang H., Yu A.C. (2019). A Biocompatible Therapeutic Catheter-Deliverable Hydrogel for In Situ Tissue Engineering. Adv. Healthc. Mater..

[30] Steele A.N., Paulsen M.J., Wang H., Stapleton L.M., Lucian H.J., Eskandari A., Hironaka C.E., Farry J.M., Baker S.W., Thakore A.D. (2020). Multi-phase catheter-injectable hydrogel enables dual-stage protein-engineered cytokine release to mitigate adverse left ventricular remodeling following myocardial infarction in a small animal model and a large animal model. Cytokine.

[31] National Research Council (US) Committee for the Update of the Guide for the Care and Use of Laboratory Animals (2011). The National Academies Collection: Reports funded by National Institutes of Health. Guide for the Care and Use of Laboratory Animals.

[32] Purcell B.P., Elser J.A., Mu A., Margulies K.B., Burdick J.A. (2012). Synergistic effects of SDF-1α chemokine and hyaluronic acid release from degradable hydrogels on directing bone marrow derived cell homing to the myocardium. Biomaterials.

[33] Stapleton L.M., Steele A.N., Wang H., Lopez Hernandez H., Yu A.C., Paulsen M.J., Smith A.A.A., Roth G.A., Thakore A.D., Lucian H.J. (2019). Use of a supramolecular polymeric hydrogel as an effective post-operative pericardial adhesion barrier. Nat. Biomed. Eng..

[34] Wang H., Paulsen M.J., Imbrie-Moore A.M., Tada Y., Bergamasco H., Baker S.W., Shudo Y., Ma M., Woo Y.J. (2020). In vivo validation of restored chordal biomechanics after mitral ring annuloplasty in a rare ovine case of natural chronic functional mitral regurgitation. J. Cardiovasc. Dev. Dis..

[35] Bennett J.W. (1973). Regional body surface area of sheep. J. Agric. Sci..

[36] Solomon S.D., Anavekar N., Skali H., McMurray J.J.V., Swedberg K., Yusuf S., Granger C.B., Michelson E.L., Wang D., Pocock S. (2005). Candesartan in Heart Failure Reduction in Mortality (CHARM) Investigators Influence of ejection fraction on cardiovascular outcomes in a broad spectrum of heart failure patients. Circulation.

[37] Camacho P., Fan H., Liu Z., He J.-Q. (2016). Large mammalian animal models of heart disease. J. Cardiovasc. Dev. Dis..

[38] Cikes M., Solomon S.D. (2016). Beyond ejection fraction: An integrative approach for assessment of cardiac structure and function in heart failure. Eur. Heart J..

[39] Suga H., Sagawa K., Shoukas A.A. (1973). Load independence of the instantaneous pressure-volume ratio of the canine left ventricle and effects of epinephrine and heart rate on the ratio. Circ. Res..

[40] Suga H., Sagawa K. (1974). Instantaneous pressure-volume relationships and their ratio in the excised, supported canine left ventricle. Circ. Res..

[41] Sagawa K., Suga H., Shoukas A.A., Bakalar K.M. (1977). End-systolic pressure/volume ratio: A new index of ventricular contractility. Am. J. Cardiol..

[42] Stone G.W., Selker H.P., Thiele H., Patel M.R., Udelson J.E., Ohman E.M., Maehara A., Eitel I., Granger C.B., Jenkins P.L. (2016). Relationship Between Infarct Size and Outcomes Following Primary PCI: Patient-Level Analysis From 10 Randomized Trials. J. Am. Coll. Cardiol..

[43] Rodell C.B., Lee M.E., Wang H., Takebayashi S., Takayama T., Kawamura T., Arkles J.S., Dusaj N.N., Dorsey S.M., Witschey W.R.T. (2016). Injectable Shear-Thinning Hydrogels for Minimally Invasive Delivery to Infarcted Myocardium to Limit Left Ventricular Remodeling. Circ. Cardiovasc. Interv..

